# Clinical and imaging differences between Stanford Type B intramural hematoma-like lesions and classic aortic dissection

**DOI:** 10.1186/s12872-023-03413-6

**Published:** 2023-07-28

**Authors:** Chuanbin Wei, Jinping Li, Enhui Du, Yulu Miao, Pinpin Li, Wenhua Guan

**Affiliations:** 1 The Third Clinical College of Xinxiang Medical University, Xinxiang, China; 2 Department of Radiology, The Third Affiliated Hospital of Xinxiang Medical University, Xinxiang, China; 3 Chinese Center for Disease Control and Prevention, Beijing, China

**Keywords:** Acute aortic syndrome, Intramural hematoma, IMH-like lesions, CT angiography

## Abstract

**Background:**

Intramural hematoma (IMH) and Aortic dissection (AD) have overlapping features. The aim of this study was to explore the differences between them by comparing the clinical manifestations and imaging features of patients with acute Stanford type B IMH-like lesions and acute Stanford type B AD (ATBAD).

**Methods:**

This study retrospectively analysed the clinical and computed tomography angiography (CTA) imaging data of 42 IMH-like lesions patients with ulcer-like projection (ULP) and 38 ATBAD patients, and compared their clinical and imaging features.

**Results:**

(1) The IMH-like lesions patients were older than the ATBAD patients (64.2 ± 11.5 vs. 50.9 ± 12.2 years, *P* < 0.001). The D-dimer level in the IMH-like lesions group was significantly higher than that in the ATBAD group (11.2 ± 3.6 vs. 9.2 ± 4.5 mg/L, *P* < 0.05). The incidence rate of back pain was significantly higher in the ATBAD group than in the IMH-like lesions group (71.1% vs. 26.2%, *P* < 0.05). (2) The ULPs of IMH-like lesions and the intimal tears of ATBAD were concentrated in zone 4 of the descending thoracic aorta. The ULPs of IMH-like lesions and the intimal tears of ATBAD were mainly in the upper quadrant outside the lumen (64.3% vs. 65.8%, *P* > 0.05). (3) The maximum diameter of the ULPs in IMH-like lesions was smaller than that of the intimal tears in ATBAD (7.4 ± 3.4 vs. 10.8 ± 6.8 mm, *P* = 0.005). The lumen compression ratio in the ULPs plane and the maximum compression ratio of the aortic lumen in the IMH-like lesions group were smaller than that in the ADBAD group (*P* < 0.05). Fewer aortic segments were involved in IMH-like lesions patients than in ATBAD patients (5.6 ± 2.2 vs. 7.1 ± 1.9 segments, *P* < 0.005). The IMH-like lesions group had less branch involvement than that of the ATBAD group (*P* < 0.001).

**Conclusion:**

The degree of intimal tears, lumen compression ratio, extent of lesion involvement, and impact on branch arteries in ATBAD are more severe than that of IMH-like lesions. But for the ULPs of IMH-like lesions and intimal tears of ATBAD, they have astonishing similarities in the location of the partition and the lumen quadrant, we have reason to believe that intimal tear is the initial factor in the pathogenesis of this kind of disease, and their clinical and imaging manifestations overlap, but the severity is different. Concerning similarities between these two conditions, these two may be a spectrum of one disease.

## Background

Acute aortic syndrome (AAS) is a group of diseases that involving the aortic wall that mainly manifest as sudden chest and back pain. It includes aortic dissection (AD), intramural hematoma (IMH), and penetrating atherosclerotic ulcer (PAU). In the general population, the incidence rate of AAS is 3.5-6.0 per 100,000 patients per year [1]. AD is the most common manifestation of AAS, accounting for 85–95% of cases, followed by IMH with 5–15% and PAU with 2–7% [2–4]. Just like AD, IMH could be divided into Stanford type A and Stanford type B, the latter accounting for 50–85% of IMH cases [5].

As early as 2010, the North American Guidelines for the Diagnosis and Management of Thoracic Aortic Diseases proposed that when the IMH term is strictly used, there is no tear or ulcer-like lesion in the intima of the aorta [6]. With the maturity of CTA technology at this stage, more intimal tears have been discovered, leading to confusion in the relationship between IMH and AD. This has raised doubts about the understanding of IMH.

Therefore, based on the current controversy, we will temporarily name these cases, which are traditionally referred to as IMH, as IMH-like lesions. This study selected the IMH-like lesions and AD cases at the medical center, both of which are Stanford B-type cases, for a more detailed comparative analysis to further explore the similarities and differences between them.

## Methods

### Research subjects

A retrospective analysis was performed on 201 patients with acute aortic syndrome who underwent thoracoabdominal CTA evaluation in our department from January 2018 to December 2022, including 92 cases of AD, 56 cases of type A AD and 36 cases of type B AD. There were 72 cases of IMH-like lesions, 30 cases of type A and 42 cases of type B IMH-like lesions and 37 cases of PAU. Inclusion criteria: (1) sudden chest pain and were diagnosed with AAS by CTA; (2) cases meeting the diagnostic criteria for type B IMH-like lesions and ATBAD; (3) complete clinical data. Exclusion criteria: (1) acute type A aortic IMH-like lesions, acute type A AD, traumatic AD and PAU (n = 125); and (2) other aortic diseases, such as Takayasu arteritis, Infectious aortitis (n = 6). At least, a total of 38 patients with ATBAD and 42 patients with IMH-like lesions were included. The protocol of this study was approved by the Ethics Review Committee of the Third Affiliated Hospital of Xinxiang Medical College (Ethical Review No.: K2022-040-01).

### Equipment and methods

All CTA scans were performed with a 64-slice spiral CT machine (GE Revolution HD, GE Healthcare, Milwaukee, USA or Somatom Definition AS+, Siemens Healthineers, Erlangen, Germany) using an automatic trigger system during the arterial and venous phases using 120 kV, 190 mAs, a collimation of 64 × 0.6 mm, a rotation time of 0.38 s. All CT scans were performed from the thoracic inlet to the level of the bifurcation of the common iliac arteries in the cranio-caudal direction, with a slice thickness of 0.625 mm and an interslice gap of 0.625 mm. Iopromide ([Ultravist® 370] Schering Pharmaceutical, Guangzhou, China), the contrast agent, was intravenously injected (concentration: 370mgI/ml, 1-2mL/kg, 4–5ml/s). When the attenuation trigger value reached a threshold of 120 Hounsfield units, a region of interest was drawn in the descending aorta, and automatic bolus tracking was done to trigger the scan.

### Data collection

#### Clinical data

The basic data of patients, including onset-admission time, number of examinations, risk factors(age, sex, hypertension, diabetes, et al.), lab test results, and surgical records, were collected through retrospective review of medical records.

#### Image data collection

All images were processed with Multimodality Workplace 2008 A (SIEMENS Medical Systems) or Advantage Workstation 4.5 (GE Medical Systems). Various image processing modalities such as multiplanar reformations (MPR), curved planar reconstructions (CPR) were used.

#### Diagnostic criteria

The diagnosis criterion of IMH-like lesions is defined by cross-sectional imaging findings, The aortic tube wall was crescents or rings with “thickening” > 5 mm, the thickened hematoma was not strengthened, the diameter of the active vessel lumen was narrowed, the inner margin of the aortic lumen was smooth, there was no free internal membrane, no true or false lumen structure, and ULP could be observed. The ulcer-like projection (ULP) was defined as a contrast agent-filled pouch protruding into the thrombosed false lumen of the aorta.

**Fig. 1:**
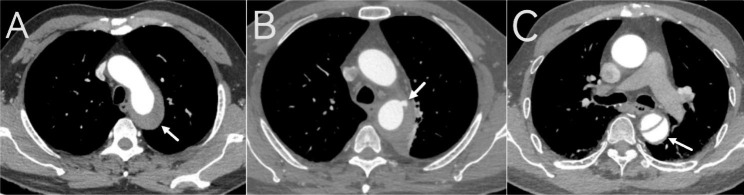
Imaging manifestations of IMH, ULP, and AD. (**A**) ATBIMH: Aortic arch and the surrounding area of the descending thoracic aorta without contrast filling. (**B**) ATBIMH around the descending thoracic aorta with ULP (white arrow). (**C**) Classical ATBAD manifestations: a free intimal flap and both true and false lumens. ATBIMH, acute type B aortic intramural haematoma; ATBAD, acute type B aortic dissection; ULP, ulcer-like projection

The diagnostic criterion of AD was a classic double-channel aorta with a visible intimal tear or flap, it could be seen the intimal flap separating true and false lumens and contrast agent filling to varying degrees in the true and false lumens (Fig. 1). Stanford type B referred to a dissection that involved the descending thoracic aorta and its distal ends beyond the left subclavian artery.

### CTA observation content

#### Intimal tear/ULP

**Fig. 2:**
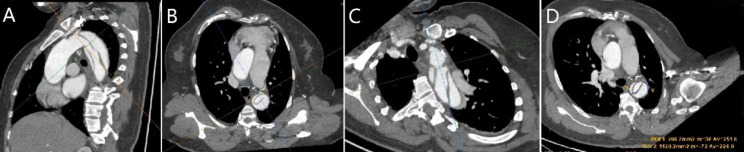
Measurement of the compression ratio of the aortic lumen using multiple planes. A 52-year-old woman presented with ATBAD. Since the intimal tear was located in the tortuosity of the aorta. The location of the intimal tear, its distance from the left subclavian artery, and the compression ratio of the aortic lumen (73.81%) were determined by three-dimensional double-oblique MPR reconstruction

**Fig. 3:**
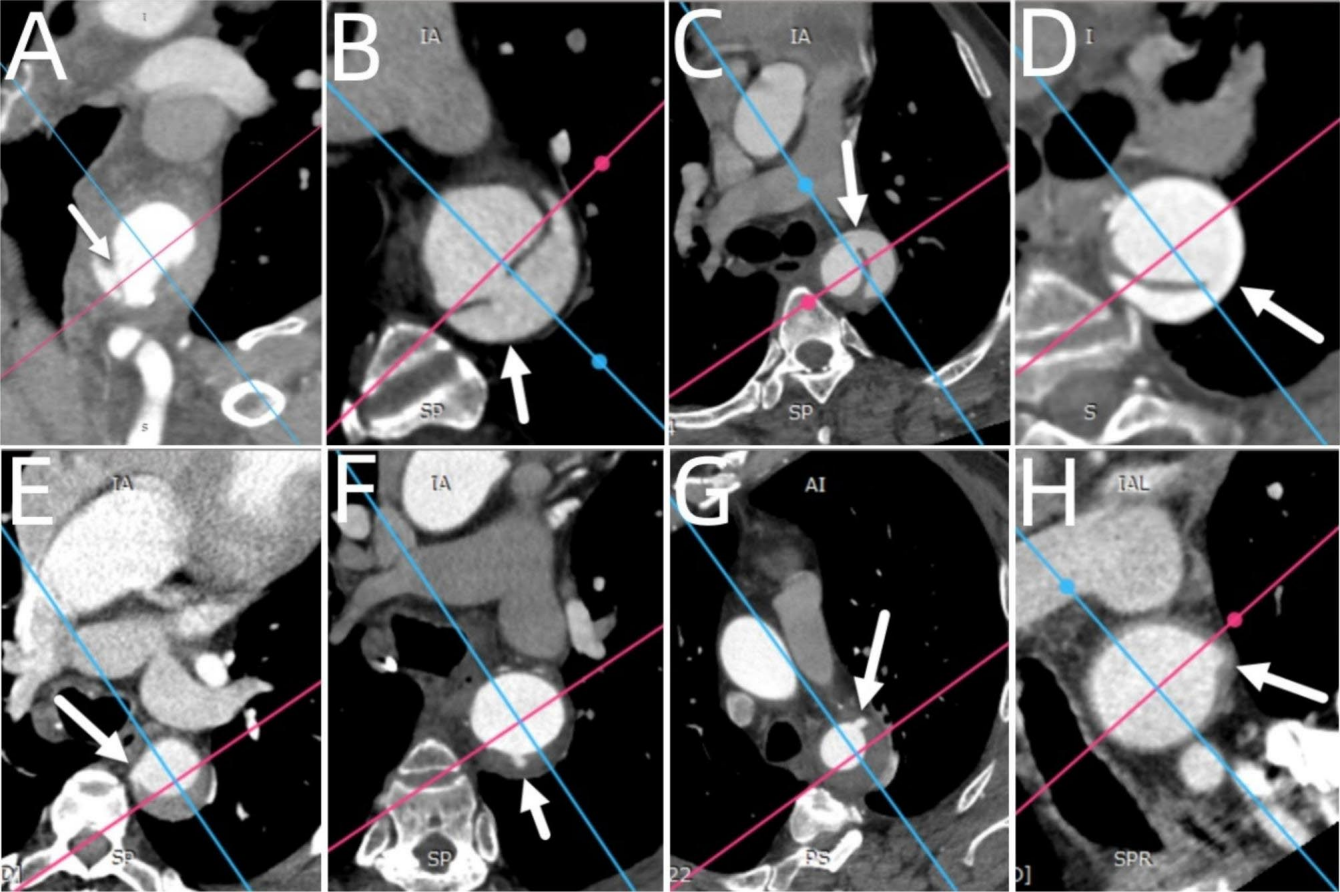
Quadrant distribution pattern of intimal tears in IMH and AD. The blue line runs along the main axis of the artery and the red line runs along the vertical line along the main axis of the artery. (**A,E**) The intimal tear was in the right quadrant. (**B,F**) The intimal tear was in the lower quadrant; (**C,G**) The intimal tear was in the upper quadrant; (**D,H**) The intimal tear was located in the left quadrant

The intimal tear/ULP location (zone, quadrant), size (maximum diameter), and distance from the left subclavian artery (Fig. 2) were measured by manual double-oblique MPR. The locations and quadrants of the intimal tears or ULPs in the two groups were obtained by MPR. The midpoint was axially placed in the centre of the aortic lumen. The long axis of the aorta and its normal line formed four quadrants (upper, lower, right, and left). For cases where the intimal tears were distributed in two quadrants, the quadrant with the highest proportion of intimal tears was selected (Fig. 3). The standard axial image was obtained by oblique MPR when the intimal tear occurred in the tortuous aortic segment.

#### Luminal involvement

The luminal involvement was assessed by the luminal compression ratio at the intimal tear/ULP, the measured maximum diameter of the descending aorta, and the extent of the lesion. We defined the lumen compression ratio as (total aortic area – cross-sectional area of the true lumen)/total aortic area. The lumen compression ratio at the aortic curvature was measured by double-oblique MPR, and the lumen compression ratios at the other parts were measured axially. The maximum diameter of the descending aorta and maximum hematoma thickness was also measured axially.

#### Lesion extent

**Fig. 4:**
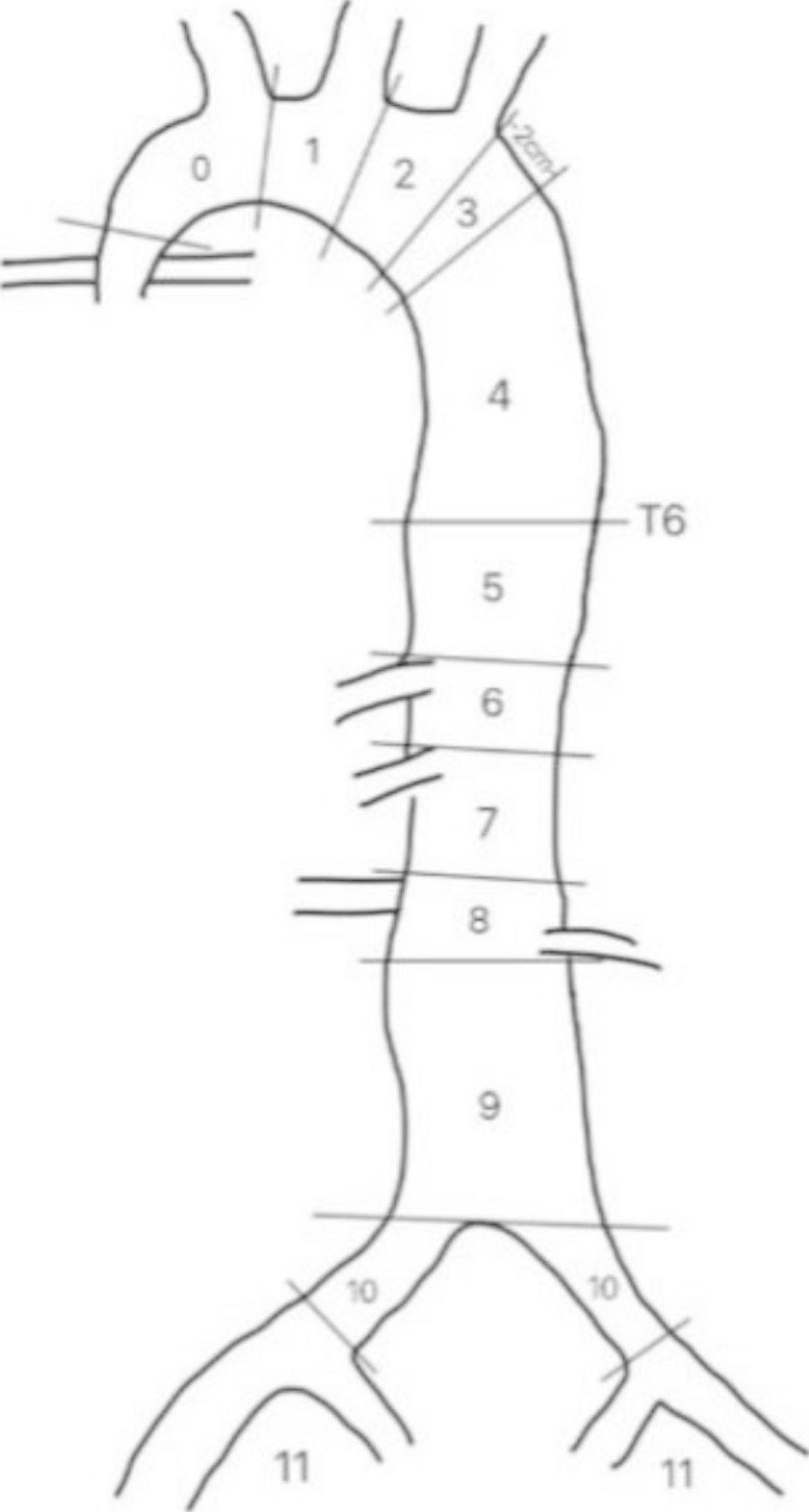
Society for Vascular Surgery and Society of Thoracic Surgeons reporting standards for type B aortic dissection

The lesion extent was determined based on the aortic division method of the Society for Vascular Surgery and the Society of Thoracic Surgeons (Fig. 4) [7].

#### Side branch malperfusion syndromes

**Fig. 5:**
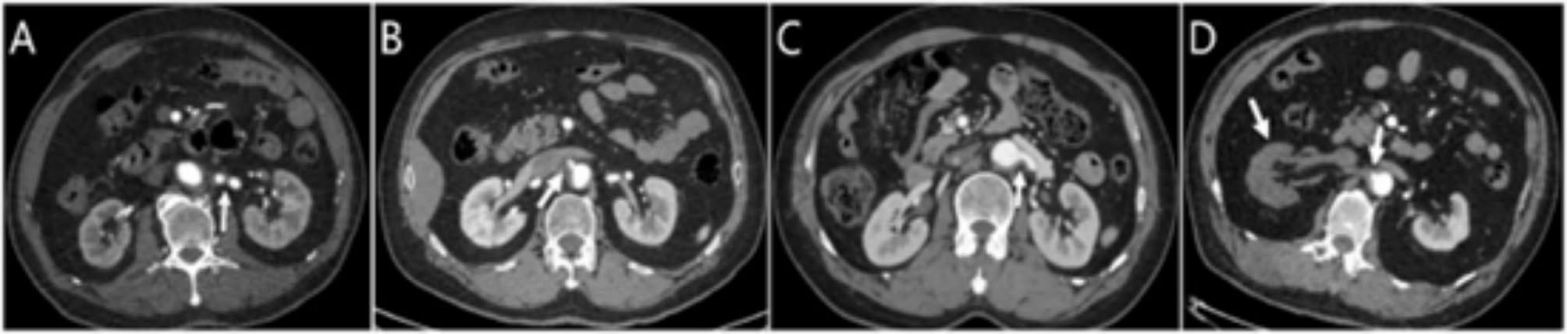
Manifestations of branch involvement and malperfusion complications in ATBIMH. **A-D** show the imaging manifestations of static obstructions caused by ATBIMH. (**A**) Haematoma involving the left renal artery and normal left renal perfusion. (**B**) Haematoma involving the right renal artery orifice, with no abnormal perfusion in the right kidney. (**C**) Haematoma involving the left renal artery orifice with normal perfusion in the left kidney. (**D**) The ruptured orifice of the right renal artery, with no ischaemic changes in the right renal artery

The branch involvement in IMH-like lesions was assessed with reference to the branch involvement criteria for AD (Fig. 5). The images were reviewed by two radiologists with more than 5 years of experience in cardiovascular imaging (C.B.W had 10 years of experience, and J.P.L had 15 years of experience), and disagreements were resolved by consultation.

### Statistical analysis

The statistical analysis was performed using IBM SPSS statistics 27 software. The t test was used to compare the age of the patients, the diameter of the intimal tear, and the distance from the tear to the left subclavian artery between the two groups, and the χ^2^ test was used to compare the locations of the tears and the involved segments between the two groups. The count data are given as n (%) and were compared between groups using the χ^2^ test or Fisher’s exact test, as needed. The quadrant distribution characteristics of intimal tears of the two groups are expressed in percentages. The interobserver agreement of quantitative data was tested by paired t test, with a confidence interval of 95% and *P* > 0.05 indicating interobserver agreement. The interobserver agreement of count data was tested by κ value, with κ ≥ 0.75 indicating excellent agreement, κ between 0.40 and 0.75 indicating medium–high agreement, and κ ≤ 0.40 indicating poor agreement. Spearman’s rank correlation coefficient analysis was used to analyse the association between the involved segment, the diameter of the tear, and the compression ratio of the aortic lumen at α = 0.05.

## Results

### Clinical features

IMH-like lesions patients were older than ATBAD patients (64.2 ± 11.5 vs. 50.9 ± 12.2years, *P* < 0.001). The proportion of male patients in the IMH-like lesions group was slightly higher than that in the ATBAD group (83.3% vs. 78.9%, *P* = 0.676). Both groups had clinical manifestations of chest pain, back pain, or abdominal pain, but the ATBAD group had a significantly higher rate of back pain than the IMH-like lesions group (71.1% vs. 26.2%, *P* = 0.002). The D-dimer level in the IMH-like lesions group was significantly higher than that in the ATBAD group (11.2 ± 3.6 vs. 9.2 ± 4.5 mg/L, *P* = 0.031). There were no significant differences in sex, hypertension, diabetes mellitus, coronary heart disease, or smoking history between the two groups. Among the risk factors, only Marfan syndrome was associated with ATBAD (Table 1).

**Table 1:** Clinical data of 42 patients with Stanford type B IMH and 38 patients with Stanford type B AD

Clinical manifestations	Type B IMH (n = 42)	Type B AD (n = 38)	t/χ²/Fisher	P
Onset-admission time (h, $\bar{x}\pm s$)	101.7 ± 80.2	5.1 ± 3.4	7.413	< 0.001^*^
Age (years, $\bar{x}\pm s$)	64.2 ± 11.5	50.9 ± 12.2	-5.047	< 0.001^*^
Sex, male/female (n, %)	35/7	30/8	0.175	0.676
Hypertension (n, %)	18(42.9)	20(52.6)	0.349	0.555
Marfan syndrome	0(0)	4(10.5)	0.213	0.051
D-Dimer (mg/L)	11.2 ± 3.6	9.2 ± 4.5	-2.201	0.031^*^
Diabetes mellitus (n, %)	4(9.5)	3(7.9)	0.622	0.430
Coronary heart disease (n, %)	7(16.7)	5(13.2)	1.507	0.22
Smoking history (n, %)	19(45.2)	18(47.4)	0.441	0.507
Clinical symptoms (n, %)				
Chest pain	15(35.7)	17(44.7)	0.01	0.919
Back pain	11(26.2)	27(71.1)	13.107	< 0.001^*^
Abdominal pain	4(9.5)	4(10.5)	-0.542	0.59
Limb ischaemia	0(0)	3(7.9)	3.445	0.103

Remarks: *, the difference between the two groups was statistically significant

### Comparison of imaging features

The maximum diameter of intimal tears in IMH-like lesions patients was smaller than that in ATBAD patients (7.4 ± 3.4 vs. 10.8 ± 6.8 mm, *P* = 0.005 < 0.05).

#### Intimal tear/ULP zones

The ULPs of IMH-like lesions and the intimal tears of ATBAD were concentrated in zone 4 of the descending thoracic aorta (the upper half of the straight segment of the descending thoracic aorta, ending at the midpoint of the descending thoracic aorta). In the IMH-like lesions group, ULPs occurred in zone 4 in 23 cases (63.9%, 23/36), in zone 3 in 11 cases (30.6%, 11/36), in zone 2 in one case (2.8%, 1/36), and in zone 5 in one case (2.8%, 1/36). In the ATBAD group, the intimal tears occurred in zone 4 in 24 cases (63.2%, 24/38), in zone 3 in 11 cases (28.9%, 11/38), in zone 2 in two cases (5.3%, 2/38), in zone 1 in one case (2.6%, 1/38), and in zone 5 in one case (2.6%, 1/38). There was no significant difference in the distribution of tear locations between the two groups (*P* = 1.000).

#### Intimal tear/ULP quadrants

The ULPs of IMH-like lesions and the intimal tears of ATBAD were mainly in the upper quadrant outside the lumen (AD: 25/38, 65.8% vs. IMH: 27/42, 64.3%). In the ATBAD group, the intimal tears were in the left quadrant in four cases (4/38, 10.5%), in the right quadrant in six cases (6/38, 15.8%), and in the lower quadrant in three cases (3/38, 7.9%). In the IMH-like lesions group, the intimal tears were in the left quadrant in one case (1/42, 2.4%), in the right quadrant in eight cases (8/42, 19.1%), and in the lower quadrant in six cases (6/42, 14.3%). There was no significant difference in the quadrant distribution of tears between the two groups (*P* = 0.294).

#### Luminal involvement

The IMH-like lesions patients had a lower compression ratio of the aortic lumen in the intimal tear plane than the ATBAD patients (48.3 ± 13% vs. 56.6 ± 18.5%, *P* = 0.031). The maximum compression ratio of the aortic lumen at the hematoma plane in the IMH-like lesions group was lower than that in the ATBAD group (48.9 ± 12.2% vs. 62.5 ± 15.9%, *P* < 0.001). There was no statistically significant difference in the maximum diameter of the descending aorta in the IMH-like lesions group and that in the ATBAD group (35.7 ± 4.3 vs. 37.7 ± 7.0 mm, *P* = 0.125 > 0.05). There was no statistically significant difference in the distance between the tear and the distal end of the left subclavian artery in the IMH-like lesions group and that in the ATBAD group (25.5 ± 20.4 vs. 22.8 ± 18.7 mm, *P* = 0.59 > 0.05).

#### Extent of lesion involvement

Patients with IMH-like lesions had fewer involved segments than patients with ATBAD (5.6 ± 2.2 vs. 7.1 ± 1.9 segments, *P* = 0.001). IMH-like lesions usually involved zones 3 to 5 of the descending aorta, whereas ATBAD usually involved zones 4 to 6 of the descending and abdominal aorta. The internal iliac artery was involved in 14 patients in the ATBAD group but was not involved in the IMH-like lesions group. The maximum intimal tear diameter and the compression ratio of the aortic lumen had no correlation with the extent of the aortic segments involved in the two groups.

#### Side branch malperfusion syndromes

There were seven cases (7/42, 16.7%) in the IMH-like lesions group of poor renal artery perfusion, including one of right renal ischaemia. There were 27 cases (27/38, 71.1%) in the ATBAD group of branch artery involvement, including one case of superior mesenteric artery involvement with partial ischaemia of the left colon, one case of bilateral internal iliac artery involvement with ischaemia manifestations in the lower extremities, one case of the left subclavian artery involvement and left limb activity disorder, and the others of renal artery involvement with or without manifestations of renal ischaemia. The interobserver agreement was excellent (κ = 0.822; 95% CI: 0.701–0.944) (Table 2).

**Table 2:** CTA findings of the 42 Stanford B IMH patients and the 38 Stanford B AD patients

Imaging features	Type B IMH (n = 42)	Type B AD (n = 38)	t/χ²/Fisher	P
Patients with intimal tears (n, %)	36(88.1)	38(100)		
Intimal tear diameter (mm, $\bar{x}\pm s$)	7.4 ± 3.4	10.8 ± 6.8	2.903	0.005^*^
Intimal tear zones (n, %)			2.008	1.000
Zone 1	0	1(2.6)		
Zone 2	0	2(5.3)		
Zone 3	11(26.2)	11(28.9)		
Zone 4	23(54.8)	24(63.2)		
Zone 5	1(2.4)	1(2.6)		
Quadrant distribution			3.791	0.294
Upper quadrant	27(64.3)	25(65.8)		
Lower quadrant	6(14.3)	3(7.9)		
Right quadrant	8(19.1)	6(15.8)		
Left quadrant	1(2.4)	4(10.5)		
Maximum diameter of the descending aorta (mm)	35.7 ± 4.3	37.7 ± 7	1.550	0.125
Maximum hematoma thickness (mm)	10.2 ± 2.6			
Luminal compression ratio in the intimal tear plane (%)	48.3 ± 13	56.6 ± 18.5	2.207	0.031^*^
Luminal compression ratio at the largest slice of the haematoma (%)	48.9 ± 12.2	62.5 ± 15.9	4. 282	< 0.001^*^
Distance between the tear and the distal end of the left subclavian artery (mm)	25.5 ± 20.4	22.8 ± 18.7	-0.542	0.590
Number of segments involved (n, $\bar{x}\pm s$)	5.6 ± 2.2	7.1 ± 1.9	40	< 0.001^*^
Pericardial effusion (n, %)	3(7.1)	2(5.3)	0.254	0.615
Pleural effusion (n, %)	8(19.1)	4(10.5)	3.673	0.055
Branch artery involvement (n, %)	7(16.7)	27(71.1)	23.808	< 0.001^*^
Organ malperfusion (n, %)	1(7.1)	11(28.9)	-3.534	<0.001^*^
Aortic rupture (n, %)	1(7.1)	0	0.916	0.338

Remarks: *, the difference between the two groups was statistically significant

## Discussion

The pathogenesis of AD is relatively clear. AD is defined as a media tear caused by unclear reasons, resulting in the dissection of various layers of the aortic wall, and medial degeneration is considered the final common pathway of different causes of AAS [8]. The formation of true and false lumens is a characteristic manifestation of AD, and the initiating factor is tearing of the intima, which enables blood to enter the intima-media space, longitudinally separating the lumen along the course of the aorta. Usually, there are one or more tears in the intima connecting the true and false lumens, and as AD progresses from acute to chronic, the false lumens exhibit a variety of morphologies [4, 9]. CTA is the preferred examination method for diagnosing AD. In the arterial phase, if the false lumen blood flow was slow and the contrast agent was not well filled or if there was a false lumen thrombosis, AD was difficult to distinguish from IMH.

Unlike AD, the pathogenesis of IMH is still unclear, and there are currently some different hypotheses [10, 11]. But one thing could be believed is that people’s understanding of IMH is more comprehensive than before. The traditional view is that IMH has been described as a noncommunicating type of dissection [12]. The hematoma was formed due to spontaneous rupture and haemorrhage of the vasa vasorum in the aortic media and expands outwards, resulting in dehiscence of the media. The hematoma is between the media and extima, the intima is not ruptured, and there is no direct communication between the hematoma and the aortic lumen [1]. However, there has been little direct clinical or experimental evidence to support the hypothesis of vasa vasorum rupture so far [11]. In the early stages, CTA examination was limited by the spatial and temporal resolutions of the equipment at that time, no intimal tear was found, so early imaging studies also agreed with the above explanation [13]. With the rapid development of multidetector computed tomography, an increasing number of “small tears” (ULPs) have been confirmed to exist in IMH. Some Japanese scholars regard IMH as a special thrombotic acute AD, also called sandwich variant IMH, which may be accompanied by intimal defects, early closure, and a thrombosed false lumen without re-entry tears [10, 14, 15]. In some reports on IMH, 36% of type A IMHs and 60% of type B IMHs are associated with intimal tears, and surgery has also confirmed that most patients with IMH have intimal tears [1, 16–19]. In the present study, 85.71% of the IMH-like lesions patients were found to have ULPs at the CTA examination, 23.81% of IMH-like lesions patients found ULP during their first CTA examination, while 76.19% of patients only showed ULP during multiple examinations. Since such ULPs continue to be discovered, it is reasonable to speculate that this type of small intimal tear may represent the initial site of injury and may be the ultimate cause of IMH rather than vasa vasorum rupture [18]. Due to the existence of intimal tearing, some scholars believe that IMH may be a precursor or a variant form of AD [11, 20]. Some 12–47% of IMH patients progress to AD, which seems to confirm the inextricable relationship between them [11].

It is precisely because of such controversy that we have named our group of cases as IMH-like lesions. In our research, we compared the clinical of ATBAD and IMH-like lesions firstly. The clinical manifestations of the two were similar, but the ATBAD group more often had back pain than the IMH-like lesions group, while the IMH-like lesions group had higher d-dimer than the ATBAD group, which has a certain significance for differential diagnosis in the clinic. IRAD and previous studies have shown that IMH mainly occurs in the elderly and is older than AD patients. In this study, the age of the IMH group was older than that of the AD group, which was consistent with previous studies [21–23].

Whether ULPs of IMH-like lesions and intimal tears of AD have similar imaging features is a focus of this study. In this study, ULPs mainly occurred in the proximal aorta in the IMH-like lesions patients, most of these in zone 4. ULPs in IMH are similar to tears in AD, but different in sizes. The degree of intimal tear of ULPs was smaller than that of AD of the same type, and the longest transverse diameter of ULPs was 7.35 ± 3.41 mm, while that of the tears in AD was 10.83 ± 6.75 mm, which is basically consistent with the results of Li et al. [24]. Drawing on previous studies on the distribution characteristics of intracranial atherosclerotic plaques on axial images [25], we used a quadrant distribution model and focused on analysing the axial distribution characteristics of two groups of intimal tears. We found that the intimal tears in both groups were mainly distributed in the upper quadrant in zone 4, which is the lateral border of the aortic wall; that is, the locations of ULPs were highly consistent with the location of the intimal tears in AD. In terms of pathogenesis, in the presence of hypertension, the shear stress generated by blood flow on the wall of the descending aorta distal to the left subclavian artery was the largest, so intimal tears were more likely to occur in this area. Therefore, ULPs can be considered highly similar to intimal tears in AD, but with different degrees of tearing. Through a more detailed analysis of the anatomical distribution of ULP, it can be inferred that the impact of blood flow on the aortic wall may not be different in IMH-like lesions and AD.

Along with intimal tears, the extent of involvement and the compression of the lumen by the false lumen or IMH also reflects the disease severity. Choi et al. [26] defined the compression ratio of the true lumen by measuring the longest and shortest transverse diameters of the true lumen and calculating the ratio between the two. They concluded that when the ratio was less than 0.75, IMH is at risk of progressing to AD, When the area of false cavity is larger, the pressure of true cavity is more obvious. Based on this, we defined the lumen compression ratio as (total aortic area – cross-sectional area of the true lumen)/total aortic area. In our study, the lumen compression ratio of ATBAD was greater than that of IMH-like lesions. The risk of IMH-like lesions during the initial examination phase was similar to that of AD, with a higher compression ratio, which also supports the fact that AD is a more severe disease.

Branch involvement of AAS can lead to poor end-organ perfusion, a major complication that should be given enough attention as an important prognostic indicator. Approximately 25% of ATBAD patients experienced side branch Malperfusion Syndromes [27]. When AD is complicated with organ malperfusion, despite optimal treatment, the mortality rate remains higher (approximately 20%) [27]. For other patients, the incidence of collateral involvement is relatively low, and there are few reports of major cases. Dreisbach et al. [28] reported the absence of branch involvement in IMH, and Evangelista et al. [29] also suggested that IMH is unlikely to be poorly perfused. But Ibukuro et al. [30] proposed that the probability of branch artery involvement in IMH was 12.9%.In our study, 7 patients with IMH-like lesions had renal artery involvement, with an incidence rate of 16.7% (7/42), which is higher than previous reports. We comparatively analysed the imaging findings of IMH patients with branch involvement and AD patients with branch involvement and found that all IMH-like lesions patients showed different degrees of renal artery involvement and that severe cases had no contrast agent in the lumen of the initial part of the right renal artery but had right renal ischaemia. We don’t yet know whether the anatomical pathology of IMH-like lesions branch involvement is similar to that of AD. Does branch involvement also support IMH as a special form of AD? Due to the involvement of the IMH branch artery, it will increase its mortality. Therefore, in the diagnosis of IMH, it is necessary to describe the branch involvement in detail as much as possible, which is of great value for prognosis judgment and surgical guidance.

Our study shows that both IMH-like lesions and ATBAD involve the thoracic and abdominal aorta to different degrees and that IMH-like lesions involves fewer aortic segments than ATBAD. IMH-like lesions usually involve zones 3 to 5 of the descending aorta, whereas ATBAD usually involves zones 4 to 6 of the descending and abdominal aorta. Furthermore, in our study, IMH-like lesions did not involve the bilateral common iliac arteries, which may indirectly explain the less effect of IMH-like lesions on branch arteries than that of AD.

Although as in our study, there are many similarities in the imaging manifestations of IMH-like lesions and AD. But it cannot fully explain that IMH-like lesions is a special type of AD. The first condition for enrolment of patients in our defined IMH-like lesions group is that clear ULP can be observed on CTA images. Past studies have confirmed that some IMH patients cannot fully observe the micro-tear through CTA examination, but the existence of the micro-tear was found during the operation. In addition, it should be noted that there is some classic dissection with acute false lumen thrombosis mixed in the IMH-like lesions case group. Although the onset time of the selected IMH-like lesions cases is 101.69 ± 80.23 h, which is much higher than the onset time of classical AD, the pseudocavitary thromboembolization of thrombotic AD can also be formed in a short time. The two cannot be completely distinguished by the onset-admission time, and their imaging manifestations are also very similar. Luca A et al. [31]in the case report on iatrogenic aortic dissection, it was suggested that during CTA, when the intimal injury produced a small channel, the contrast medium would enter the aortic wall, and once the channel was closed, it would reexamine the appearance of aortic intramural hematoma on PCI a few days later. As Terrence D et al. [32] said, iatrogenic AD often occurs in the form of IMH and may be a more focal process.

The natural outcome of IMH lesions is different from that of AD. It can be absorbed without intervention, or it may develop into a typical aortic dissection or rupture, about 12–47% of IMH patients progress to AD [11, 20]. Because AD has at least two intimal lacerations, thus relieving the pressure in the aortic false lumen. What’s different is that most IMH have only one intimal tear, and the aortic wall may be subjected to greater pressure, making it more likely that IMH will develop into AD or rupture [4, 33]. Alomari et al. [20] study showed that about 15% of IMH patients developed aortic rupture, and 3% of ATBIMH patients and 88% of ATAIMH patients developed acute aortic dissection. More detailed histopathological studies, Uchida et al. [34] proved that compared with AD, the dissection of IMH was closer to the lateral membrane of the aortic wall, indicating that the lumen wall of IMH was thinner than that of AD, which led to a higher risk of rupture of IMH than AD. In this study, we observed a rupture in a patient with IMH-like lesions. The main imaging findings were mediastinal hemorrhage, pericardial hemorrhage, and pleural hemorrhage. Although the probability of rupture of ATBIMH is less than that of ATAIMH [35], it is still a point worthy of our attention.

This study has several limitations. Firstly, we only observed the clinical and imaging features of the two groups of patients, and further follow-up is needed to compare the differences in prognosis between the two groups of patients. Secondly, this is a single center retrospective study, so selection bias cannot be ruled out. Thirdly, this study is based on non-electrocardiogram gated scans. Although the ascending aorta does not need to be considered in our study subjects, there is still a possibility of missed and misdiagnosis of IMH-like lesions with small ULP.

## Conclusion

The degree of intimal tears, lumen compression ratio, extent of lesion involvement, and impact on branch arteries in ATBAD are more severe than that of IMH-like lesions. But for the ULPs of IMH-like lesions and intimal tears of ATBAD, they have astonishing similarities in the location of the partition and the lumen quadrant, we have reason to believe that intimal tear is the initial factor in the pathogenesis of this kind of disease, and their clinical and imaging manifestations overlap, but the severity is different. Concerning similarities between these two conditions, these two may be a spectrum of one disease.

## Data Availability

The datasets generated and analyzed during the current study are not publicly available due to patients’ privacy but are available from the corresponding author on reasonable request.

## Abbreviations

ATBIMH: Stanford acute type B aortic intramural haematoma
ATBAD: Stanford acute type B aortic dissection
ULP: Ulcer-like projections
MPR: Multiplanar reconstruction
CPR: Curved planar reformation
CTA: Computed tomography angiography
